# Endometrial Receptivity Profile in Patients with Premature Progesterone Elevation on the Day of hCG Administration

**DOI:** 10.1155/2014/951937

**Published:** 2014-04-28

**Authors:** Delphine Haouzi, Laurence Bissonnette, Anna Gala, Said Assou, Frida Entezami, Hélène Perrochia, Hervé Dechaud, Jean-Noel Hugues, Samir Hamamah

**Affiliations:** ^1^CHU Montpellier, Institut de Recherche en Biothérapie, Hôpital Saint-Eloi, 34295 Montpellier, France; ^2^INSERM U1040, Hôpital Saint-Eloi, 34295 Montpellier, France; ^3^Université Montpellier 1, UFR de Médecine, Equipe “Développement Embryonnaire Précoce et Cellules Souches Embryonnaires Humaines”, 34000 Montpellier, France; ^4^OVO Fertility, 8000 Boulevard Decarie No. 100, Montréal, QC, Canada H4P 2S4; ^5^CHU Montpellier, ART/PGD Division, Département de Biologie de la Reproduction, Hôpital Arnaud de Villeneuve, 34295 Montpellier, France; ^6^Laboratoire Dynabio, Polyclinique du Cotentin, 50120 Equeurdreville, France; ^7^CHU Montpellier, Hôpital Gui de Chauliac, Service Anatomie Cytologie Pathologiques, 34295 Montpellier, France; ^8^CHU Léonard de Vinci-Université Paris XIII, Service de Médecine de la Reproduction, Hôpital Jean Verdier, 93143 Bondy, France

## Abstract

The impact of a premature elevation of serum progesterone level, the day of hCG administration in patients under controlled ovarian stimulation during IVF procedure, on human endometrial receptivity is still debated. In the present study, we investigated the endometrial gene expression profile shifts during the prereceptive and receptive secretory stage in patients with normal and elevated serum progesterone level on the day of hCG administration in fifteen patients under stimulated cycles. Then, specific biomarkers of endometrial receptivity in these two groups of patients were tested. Endometrial biopsies were performed on oocyte retrieval day and on day 3 of embryo transfer, respectively, for each patient. Samples were analysed using DNA microarrays and qRT-PCR. The endometrial gene expression shift from the prereceptive to the receptive stage was altered in patients with high serum progesterone level (>1.5 ng/mL) on hCG day, suggesting accelerated endometrial maturation during the periovulation period. This was confirmed by the functional annotation of the differentially expressed genes as it showed downregulation of cell cycle-related genes. Conversely, the profile of endometrial receptivity was comparable in both groups. Premature progesterone rise alters the endometrial gene expression shift between the prereceptive and the receptive stage but does not affect endometrial receptivity.

## 1. Introduction

The impact of premature serum progesterone elevation at the end of the follicular phase under controlled ovarian stimulation (COS) cycle for* in vitro* fertilization (IVF) is still debated. While several studies reported lower pregnancy rates in patients with high progesterone concentration on the day of human chorionic gonadotropin (hCG) administration [1–9], one found a favourable effect on pregnancy outcome [10] and others failed to demonstrate any association [11–21]. Although the mechanism by which premature serum progesterone elevation might alter the embryo transfer outcome is still unclear, there are accumulated data suggesting a negative impact on endometrium [22, 23]. Elevated progesterone levels might induce premature endometrial maturation and, as a consequence, earlier opening of the implantation window that leads to asynchronization of the crosstalk between embryo and endometrium. Accelerated endometrial maturation following COS has been clearly demonstrated by histological dating on the day of oocyte retrieval [24–27], but this is not the case during the implantation window [22]. Therefore, to what extent endometrial receptivity is impaired in patients with high serum progesterone level is questionable.

In addition, very few studies have assessed the impact of serum progesterone elevation on the endometrial gene expression profile during the implantation window [22, 23] (Table 1). By comparing the endometrial gene expression profiles during the implantation window in patients with high and normal serum progesterone level on the day of hCG administration, these authors found significant differences in the expression of genes that play a crucial role in endometrial function. Although some of these genes were related to endometrial receptivity, no clear assessment of the endometrial status during the implantation window in the patients with high serum progesterone level on hCG day was carried out.

The aim of the study was (i) to compare individually the endometrial gene expression shift between the prereceptive and receptive secretory stages in patients with normal (<1.5 ng/mL) and high (>1.5 ng/mL) serum progesterone levels on the day of hCG administration and, then, (ii) to test biomarkers of endometrial receptivity in these two groups of patients.

## 2. Materials and Methods

### 2.1. Patients' Characteristics and Endometrial Biopsies

The study population included 15 patients (age 31 years ±3) who were referred for intracytoplasmic sperm injection (ICSI) for male infertility and were recruited after written informed consent. This project was approved by the Ethical Committee of the Institut de Recherche en Biothérapie. All patients had normal serum FSH, LH, and estradiol on day 3 of COS under either GnRH agonist long or antagonist protocols, as well as on the day of hCG administration (Table 2). An endometrial biopsy was obtained on the day of oocyte collection (hCG+2) and another one during embryo transfer (hCG+5), respectively. Endometrial biopsies (*n* = 30) were washed and frozen individually at −80°C prior to total RNA extraction with the RNeasy Mini Kit (Qiagen, Valencia, CA, USA).

### 2.2. Progesterone Measurement

Serum progesterone was measured on the day of hCG administration by using an automated Cobas e411 instrument (Roche Diagnostics). Intra-assays and interassay coefficients of variation (CV) were <2.7% and <9.1%, respectively.

### 2.3. Microarray Hybridization

Total RNA (100 ng) was used to prepare twice amplified and labelled cRNA for hybridization with HG-U133 plus 2.0 arrays (Affymetrix, UK) as described in [29]. Each endometrial sample was processed individually on a separate DNA microarray chip.

### 2.4. Data Processing and Microarray Data Analysis

Scanned GeneChip images were processed using the Affymetrix GCOS 1.4 software to obtain the intensity value signal and the absent/present detection call for each probe set using the default analysis settings and global scaling as first normalization method. Probe intensities were derived using the MAS5.0 algorithm.

Patients (*n* = 15) were divided into two groups according to their serum progesterone concentration ([P]) on the day of hCG administration: <1.5 ng/mL (normal [P] group, *n* = 7 patients) and >1.5 ng/mL (high [P] group, *n* = 8 patients) (Table 2). The number of patients under GnRH long agonist protocol was similar in each group (2 per group). To compare the endometrial gene expression profile shift between hCG+2 and hCG+5 samples in the two groups of patients, a probe set selection using the detection call (present in all samples of the selected group) and a coefficient of variation ≥40% between endometrial samples were first carried out. Then, the significant analysis of microarrays (SAM; Stanford University) [30] was used to identify genes that were significantly differentially expressed between the hCG+2 and hCG+5 endometrium samples (paired-sample analysis) from the normal and high [P] groups. The lists of identified genes (fold change, FC > 2; false discovery rate, FDR < 5%) were submitted to Ingenuity (http://www.ingenuity.com) to identify the biological pathways/functions that were specific of the high serum [P] group. Unsupervised hierarchical clustering analyses were performed with the Cluster and TreeView software packages.

### 2.5. Quantitative RT-PCR Analyses

To assess biomarkers of endometrial receptivity, RNA (0.5 *μ*g) of receptive endometrium samples from patients with normal (hCG+5, *n* = 3) and high [P] (hCG+5, *n* = 3) on the day of hCG administration was used for reverse transcription-quantitative polymerase chain reaction (RT-qPCR) according to the manufacturer's recommendations (Applied Biosystems, Villebon sur Yvette, France). To validate some microarray data comparing the endometrial gene expression shift between prereceptive and receptive secretory stages, RNA (0.5 *μ*g) of prereceptive samples from patients with normal (hCG+2, *n* = 3) and high [P] (hCG+2, *n* = 3) was also used. For qPCR, 2 *μ*L (of a 1 : 5 dilution) of the first strand DNA was added to a 10 *μ*L reaction mixture containing 0.25 *μ*M of each primer and 5 *μ*L of 2X LightCycler 480 SYBR Green I Master mix (Roche, Mannheim, Germany). DNA was amplified during 45 cycles with annealing temperature at 63°C using the Light Cycler 480 detection system (Roche). The sample values were normalized to* PGK1* (phosphoglycerate kinase 1) expression using the following formula: *E*_tested  primer_^ΔCt^/*E*_*PGK*1_^ΔCt^  (*E* = 10^−1/slope^), ΔCt = Ct control—Ct unknown, where *E* corresponds to the efficiency of the PCR reaction. The *E* value was obtained by a standard curve that varies in function of the primers used. One receptive endometrium sample from a patient with normal serum [P] was used as control. Each sample was analysed in duplicate and multiple water blanks were included.

### 2.6. Statistical Analyses

Statistical analyses of the clinical and qRT-PCR data were performed using the GraphPad InStat 3 software. Differences between groups were considered significant when Student's *t*-test gave a *P* value < 0.05.

## 3. Results

### 3.1. COS Parameters and Embryo Transfer Outcome

COS parameters and embryo transfer outcome were not significantly different between groups but for the serum [P] on the day of hCG administration (Table 2).

### 3.2. Gene Expression Profile Shifts between the Prereceptive and Receptive Secretory Stage in the Normal and High Serum [P] Groups

**Figure 1:**
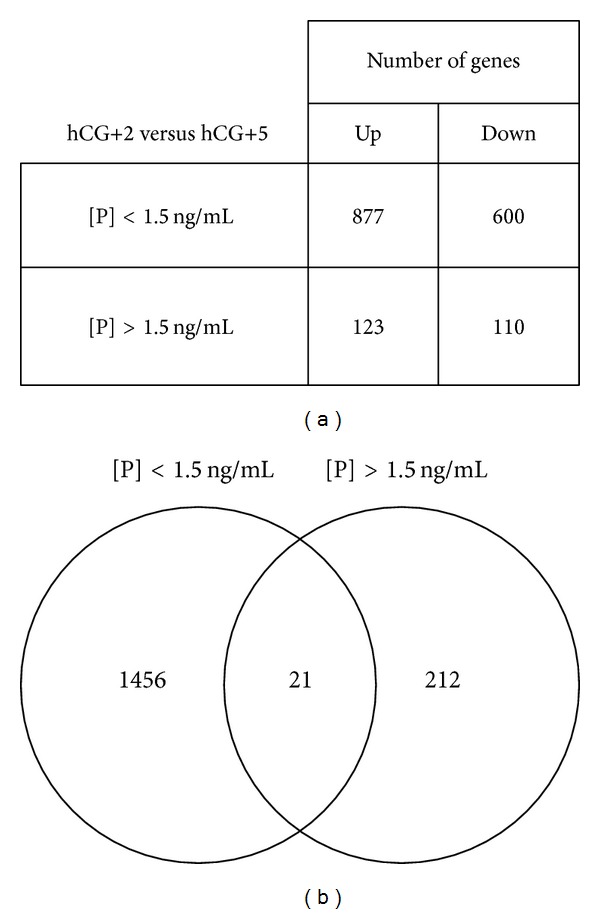
(a) Number of genes that were up- or downregulated in the normal and high [P] groups. (b) Venn diagram of the transcripts that were differentially expressed in the prereceptive and the receptive endometrial samples from patients with normal or high serum [P].

Using the detection call and the variation coefficient for a first selection of genes expressed in the hCG+2 and hCG+5 endometrium samples, 6084 and 6130 genes were identified in the normal and high [P] groups, respectively. Then, SAM analysis of these microarray data identified 1477 and 233 genes that were differentially modulated between the two endometrial stages in the normal and high [P] group, respectively. The proportion of upregulated (59% in the normal serum group and 53% in the high serum [P] group) and downregulated genes (41% in the normal serum group and 47% in the high serum [P] group) was similar in the two groups of patients (Figure 1(a)). However, the fold changes were more important in the normal group [P] (−48.9 ≤ fold change ≤ 79.9) than in the high [P] group (−5.9 ≤ fold change ≤ 40.4).

### 3.3. Cell Cycle-Related Genes Are Downregulated in the High Serum [P] Group

**Figure 2:**
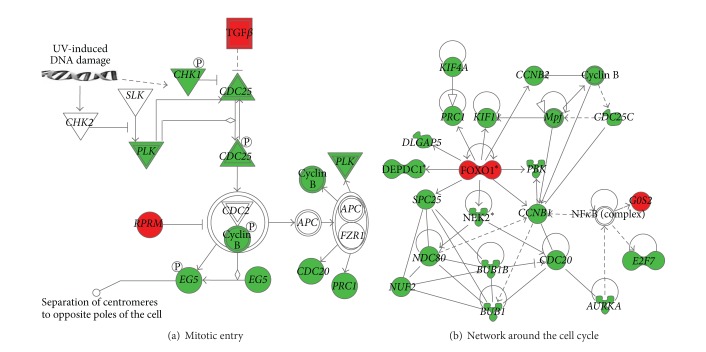
Signalling pathways that were altered in the high serum [P] group. The Ingenuity Pathway software was used to identify the functional pathways associated with genes that were differentially expressed between the prereceptive and receptive endometrial stages in the high [P] group. The majority of genes related to mitotic entry (a) and cell cycle (b) were downregulated. In this network, edge types are indicatives: a plain line indicates direct interactions, a dashed line indicates indirect interactions, a line without arrowhead indicates binding only, a line finishing with a vertical line indicates inhibition, and a line with an arrowhead indicates “acts on”. Green, downregulated; red, upregulated.

**Figure 3:**
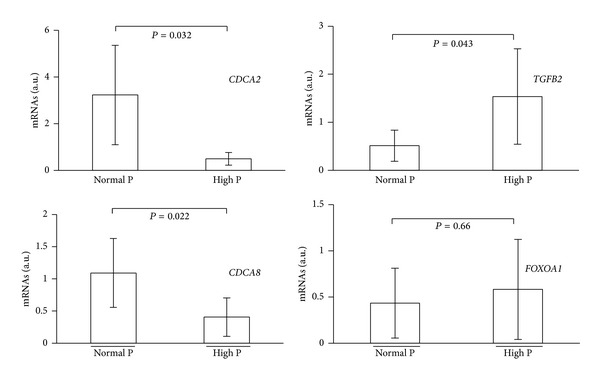
Validation by quantitative RT-PCR analysis of genes related to the cell cycle function modulated in the high [P] group. RNA isolated from hCG+2 and hCG+5 endometrial samples of normal and high serum [P] patients (*n* = 3/each group) was used. Data are the mean ± SEM. NS, nonsignificant.

To identify endometrial genes that were specifically modulated in the high serum [P] group between the prereceptive (hCG+2) and receptive (hCG+5) stage, we crossintersected the lists of genes that were differentially expressed between the two stages in the endometrium samples from the high (*n* = 233 genes) and normal (*n* = 1477 genes) [P] groups (Figure 1(b)). We identified 212 genes that were exclusively modulated in the high [P] group. Among them, more than 50 genes were involved in the cell cycle [*P* value = 2.22* E* –11 – 2.41* E* −02], including several members of the cell division cycle family (*CDC20, CDC25C, CDCA1, CDCA2, CDCA5, CDCA8*), cyclins (*CCNB1, CCNB2*), and kinesins (*KIF4A, KIF11, KIF15, KIF23*) (Figure 2 and Table S1 for the complete list) (see Table S1 in the Supplementary Material available online at http://dx.doi.org/10.1155/2014/951937); 75% of these genes were downregulated. Some of these genes (*CDCA2*,* CDCA8*,* FOXOA1,* and* TGFB2*) have been validated by qRT-PCR (Figure 3). In addition, we identified 21 genes that were common to both groups (Table 3).

### 3.4. Endometrial Receptivity in Patients with High Serum Progesterone Level

**Figure 4:**
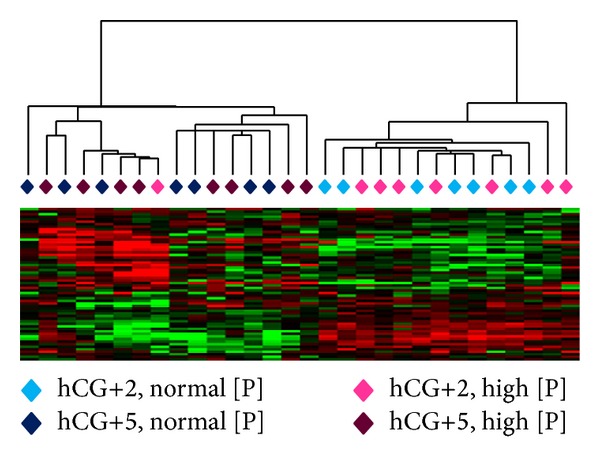
Unsupervised hierarchical clustering of the prereceptive and receptive endometrium samples using the previously described [28] predictor list. Comparison of the gene expression profiles at hCG+2 and hCG+5 in the normal and high serum [P] groups revealed similar transcriptomic profiles. Green, downregulated; red, upregulated.

To assess whether endometrial receptivity was affected in the high serum [P] group, we used the endometrial receptivity predictor list (54 genes) that we previously described [28] for unsupervised clustering of the endometrial gene expression profiles at hCG+2 (prereceptive) and hCG+5 (receptive stage). A clear segregation of the hCG+2 and hCG+5 endometrium samples independently of serum [P] was observed, suggesting a similar transcriptomic shift of endometrial receptivity biomarkers in the two groups (Figure 4).

**Figure 5:**
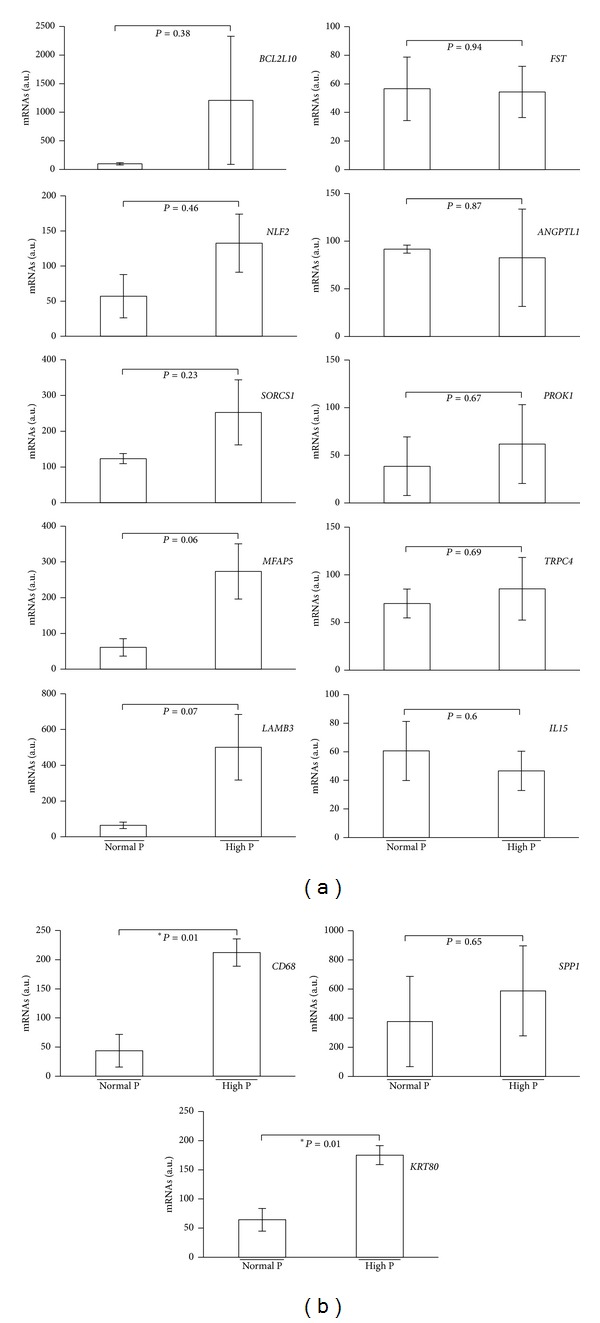
Validation by quantitative RT-PCR analysis of several biomarkers of endometrial receptivity using RNA isolated from hCG+5 endometrial samples of normal and high serum [P] patients (*n* = 3/each group). Data are the mean ± SEM. *P value < 0.05; NS, nonsignificant.

The most overexpressed predictors (*n* = 13) of the endometrial receptivity were selected for validation by qRT-PCR. No significant difference in the two groups of patients (normal versus high [P]) was observed, except for* CD68* and* KRT80* (Figure 5).

## 4. Discussion

Our data indicate that premature progesterone elevation alters the endometrial gene expression shift from the prereceptive to the receptive stage. Indeed, the transcriptomic endometrial gene expression shift in the normal serum [P] group was comparable to the one previously described for patients in COS protocols [29]. In the high serum [P] group, this transcriptomic shift was reduced with only 233 genes differentially expressed between hCG+2 and hCG+5. This finding suggests that in the high [P] group endometrial maturation is accelerated during the early secretory phase. This hypothesis was confirmed by the functional annotation analysis as it revealed that many of the downregulated genes are involved in cell cycle functions. Previous studies [24, 27] also showed an advanced endometrial maturation (2 to 4 days) based on histological dating on the day of oocyte retrieval (hCG+2). However, this was irrespective of the serum [P] on the day of hCG administration. Furthermore, it is well known that progesterone might inhibit the normal endometrial proliferation [31]. More precisely, influence of progesterone on cell proliferation appears dose dependent. Consequently, this can explain the downregulation of genes related to the cell cycle functions in patients with high serum [P]. This finding was in conjunction with functional annotations reported by Labarta et al. [22] revealing alteration of the cellular growth and proliferation in patients with high serum progesterone level under COS. Simultaneous to its critical role in the control of the proliferation status, progesterone is necessary to the acquisition of the decidualization morphotype, a key event for acquisition of the receptive endometrial status and, therefore, successful implantation.

However, alteration of the endometrial transcriptome shift in patients with high serum [P] did not seem to affect endometrial receptivity. Indeed, we observe by quantitative RT-PCR analysis that most endometrial receptivity biomarkers (genes that are upregulated during the implantation window) display similar or higher expression levels in patients with high [P] in comparison to women with normal serum [P]. These biomarkers were selected based on our previous study that identified new biomarkers of human endometrial receptivity by comparing the endometrial gene expression profile shift during the prereceptive and receptive secretory stages in the same patients during a natural cycle [28]. Two of them (*IL15* and* SPP1*) were identified by all six transcriptomic studies that compared the same endometrial samples from women in natural cycles [32].

Labarta et al. [22] examined 25 windows of implantation genes that are strongly related to endometrium receptiveness and embryo implantation and were previously described in [33]. However, only 8 of these genes were referred in their genomic diagnostic tool as specific to endometrial receptivity [34]. Based on the expression of these eight biomarkers, they concluded that endometrial receptivity was affected in patients with high serum [P] [22]. However, by thoroughly analysing their data, we think that the expression of these eight biomarkers was increased in both fertile women with natural cycles and in patients with high serum [P], albeit to a lesser extent, thus strongly suggesting that endometrial receptivity was not affected in patients with high serum [P]. In addition, using the approach described by Labarta et al. [22], we found no significant differences in the gene expression profiles of receptive endometrium samples from patients with high or normal serum [P], whatever the [P] threshold on the day of hCG administration (data not shown), thus strongly suggesting again that endometrial receptivity is similar in patients with normal and high [P].

Therefore, our findings point to an abnormally accelerated endometrial maturation but only during the prereceptive secretory phase and not during the implantation window. Consequently, transfer of a day-3 embryo in such too precociously mature endometrium would not allow the proper establishment of the embryo-endometrium crosstalk; this might explain why the pregnancy outcome was impaired when embryo transfer was performed on day 3 (hCG+5) in patients with high serum [P] on the day of hCG administration [35]. On the other hand, when embryo transfer was performed on day 5 (hCG+7), no detrimental effect on the pregnancy outcome was observed [35, 36], particularly in patients in GnRH antagonist protocols [36]. Another study reported increased cumulative pregnancy rates in patients with high serum [P] in GnRH antagonist protocols following fresh or frozen-thawed embryo transfer [37]. All these findings suggest that (i) the periovulatory endometrial maturation advancement does not necessarily lead to a deficit of endometrial receptivity and (ii) the endometrium can recover from exposure to supraphysiologic steroid concentrations [35]. Moreover, as described in Labarta et al. [22], histological dating (Noyes' criteria, [38]) revealed the absence of endometrial maturation advancement during the implantation window (hCG+7) in patients under COS protocols, regardless of the used GnRH analogues and the serum [P] on the day of hCG administration. In addition, premature elevation of serum [P] on the day of hCG administration during COS did not impact the pregnancy rate in oocyte donation programmes, suggesting a nondeleterious effect of premature [P] rise on endometrial receptivity [39, 40].

The mechanisms explaining serum [P] elevation at the time of hCG administration remain unclear. However, there are accumulating data suggesting that the main factors associated with increased risk of progesterone rise during COS cycles are ovarian parameters, including the total FSH dose, the intensity of the ovarian response, and excess number of follicles or oocytes [41]. Such parameters were not significantly different in our two groups of patients probably due to the small patients' number (Table 1). In addition, the deleterious effect of premature progesterone rise is probably not due to an impact on endometrial receptivity or ovarian parameters but rather to a desynchronized dialogue between embryo and endometrium. This hypothesis should be confirmed.

In conclusion, the gene expression profiles of the endometrial shift from the prereceptive to the receptive secretory stage are altered in patients with high serum [P] on the day of hCG administration in comparison to patients with normal [P]. This alteration suggests an acceleration of endometrial maturation during the periovulatory phase that should desynchronize the embryo-endometrium dialogue. On the other hand, endometrial advancement seems to decrease during the perireceptive phase and it does not affect endometrial receptivity.

## Supplementary Material

Supplementary Table S1: List of cell cycle-related genes that were differentially expressed in the high [P] group between the pre-receptive and the receptive endometrial stages.

## Figures and Tables

**Table 1:** Design of three microarray-based studies that investigated the impact of high serum progesterone level on the endometrial gene expression profile.

Study	Study population	Number of patients	Number of samples	Sampling time	[P] cutt-off (ng/mL)	Compared samples		Fold change	Number of genes	
									Up	Down
[22]	Oocyte donors	12	12	hCG+7	1.5	hCG+7, [P] > 1.5 versus hCG+7, [P] < 1.5		≥2	64	76
						(*n* = 6)	(*n* = 6)			

[23]	Patients undergoing IVF due to tubal or male infertility	10	10	hCG+8	1.2, 1.9	hCG+8, [P] < 1.2 versus hCG+8, [P] > 1.9		≥2	13	9
						(*n* = 5)	( (*n* = 5)			

[27]	Not specified	14	14	hCG+2	1, 1.5	hCG+2, [P] ≤ 0.9 versus hCG+2, 1 > [P] > 1.5		≥1.4	5*	23*
						(*n* = 3)	( (*n* = 6)			
						hCG+2, 1 > [P] > 1.5 versus hCG+2, [P] > 1.5			607*	212*
						(*n* = 6)	( (*n* = 5)			

This study	Normoovulatory women referred for ICSI	15	30	hCG+2, hCG+5	1.5	hCG+2 [P] < 1.5 versus hCG+5 [P] < 1.5		>2	877	600
						(*n* = 7)	( (*n* = 7)			
						hCG+2 [P] > 1.5 versus hCG+5 [P] > 1.5			123	110
						(*n* = 8)	( (*n* = 8)			

*Data are expressed in number of probe sets; *n*: number of samples; ICSI: intracytoplasmic sperm injection.

**Table 2:** Patients' clinical characteristics on the day of hCG administration and pregnancy outcome.

	[P] <1.5 ng/mL (*n* = 7)	[P] >1.5 ng/mL (*n* = 8)	*P* value
Age (years)	31 ± 4.9	30.1 ± 2.7	NS
[P] (ng/mL)	0.95 ± 0.24	2.6 ± 0.91	<0.001
E2 (pg/mL)	2509 ± 1357	2682 ± 2098	NS
LH (mIU/mL)	1.19 ± 0.19	1.25 ± 0.96	NS
Total FSH dose (IU)	1830 ± 414	1962 ± 262	NS
Number of retrieved oocytes	11.6 ± 5.2	15.5 ± 7.6	NS
Pregnancy (%)*	28.6	12.5	NS

Data are the mean ± SEM. NS: nonsignificant. *According to the serum *β*-human chorionic gonadotrophin measured 16 days after embryo transfer.

**Table 3:** List of genes shared by the normal serum [P] and high serum [P] groups.

Gene symbol	[P] <1.5 ng/mL		[P] >1.5 ng/mL	
	Fold change	FDR (%)	Fold change	FDR (%)
ATOH8	6.74	0.41	2.65	3.20
DLGAP1	2.15	0.98	3.08	4.98
GGT1	3.23	4.93	2.82	3.20
MAOA	3.41	1.48	3.23	4.98
ITGB4	3.82	0.15	2.86	3.20
MT1H	4.57	0.41	12.97	1.75
EDD1	−2.31	0.17	−2.05	0.00

INDO	−2.71	0.00	4.02	0.00
KIAA0703	−2.75	0.00	2.75	1.75
RGS16	−4.53	0.00	2.36	4.36
RGS4	−3.70	0.00	2.10	4.36
TACC2	−2.43	0.11	2.22	3.74
TNFAIP3	−2.04	0.00	2.51	3.20
MPHOSPH1	2.06	0.61	−2.09	2.91
ARHGAP26	3.00	1.74	−2.01	2.09
CCNA2	2.13	3.68	−2.09	2.91
CDC2	2.59	1.74	−2.15	2.91
DNAJC9	8.41	0.98	−2.07	0.00
FGF13	2.35	0.26	−2.18	0.00
FN1	2.33	0.15	−2.01	3.30
GPR64	2.53	1.48	−3.20	2.09
